# Progression of Discoid Lupus to Lupus Panniculitis: An Unexplained and Likely Underreported Phenomenon

**DOI:** 10.7759/cureus.9904

**Published:** 2020-08-20

**Authors:** Christopher White, David Baltazar, Richard Miller

**Affiliations:** 1 Dermatology, Hospital Corporation of America Healthcare/University of South Florida Morsani College of Medicine Graduate Medical Education: Largo Medical Center, Largo, USA; 2 Dermatology, HonorHealth Medical Center, Scottsdale, USA

**Keywords:** cutaneous lupus erythematosus, discoid lupus erythematosus (dle), lupus erythematosus profundus, systemic lupus erythema

## Abstract

Lupus erythematosus is a multiorgan disorder with a wide variance of clinical presentations. Disease processes are generally divided into systemic or cutaneous categories, with cutaneous findings being further subdivided into acute, subacute, and chronic variants. The chronic form of cutaneous lupus itself has multiple subsets. We present the case of a young woman who developed two forms of chronic cutaneous lupus erythematosus (CCLE) and, eventually, progressive systemic symptoms.

## Introduction

Cutaneous symptoms develop in approximately 80% of patients with systemic lupus erythematosus (SLE), and will be the initial manifestation of disease in 23%-28% of cases [1]. Moreover, the diagnosis of cutaneous lupus portends a 20% probability of developing systemic manifestations within three years [2]. 

Acute cutaneous lupus erythematosus (ACLE) is most notably identified as a transient rash of the malar face, classically referred to as a “butterfly” rash; approximately 95% of those with ACLE will eventually meet criteria for SLE [1]. Beginning as erythematous to edematous macules and papules on the central face with characteristic sparing of the nasolabial folds, these findings may coincide with the diagnosis of systemic lupus or precede development by weeks to years [1,3]. Erosions and ulcerations of the affected areas may develop, while a generalized photosensitive dermatitis may serve as a harbinger of multiorgan disease.

Subacute cutaneous lupus erythematosus (SCLE) begins as photodistributed macules and papules that evolve into annular (42%) or psoriasiform (39%) plaques, with 16% of cases displaying a morphologic overlap of these two findings [3]. While lesions often leave hypopigmentation with resolution, they heal without scarring [4]. SCLE precedes the diagnosis of SLE in up to 50% of patients but is widely linked to milder systemic disease, with <10% developing severe symptoms [1,3,4]. There have also been more than 40 drugs linked to drug-induced SCLE, making it the most common variant of cutaneous lupus to be caused by medication [2].

Chronic cutaneous lupus erythematosus (CCLE) is an intensely inflammatory process with subtypes, including discoid lupus erythematosus (DLE) and lupus panniculitis (LP). Lupus erythematosus tumidus and chilblain lupus are generally accepted to be variants of CCLE, but lack many of the characteristic findings seen in other forms and, thus, are outside the scope of this review [1-3]. CCLE foreshadows the lowest probability of developing SLE, with 5%-20% of patients going on to meet the diagnostic criteria [3].

## Case presentation

**Figure 1:**
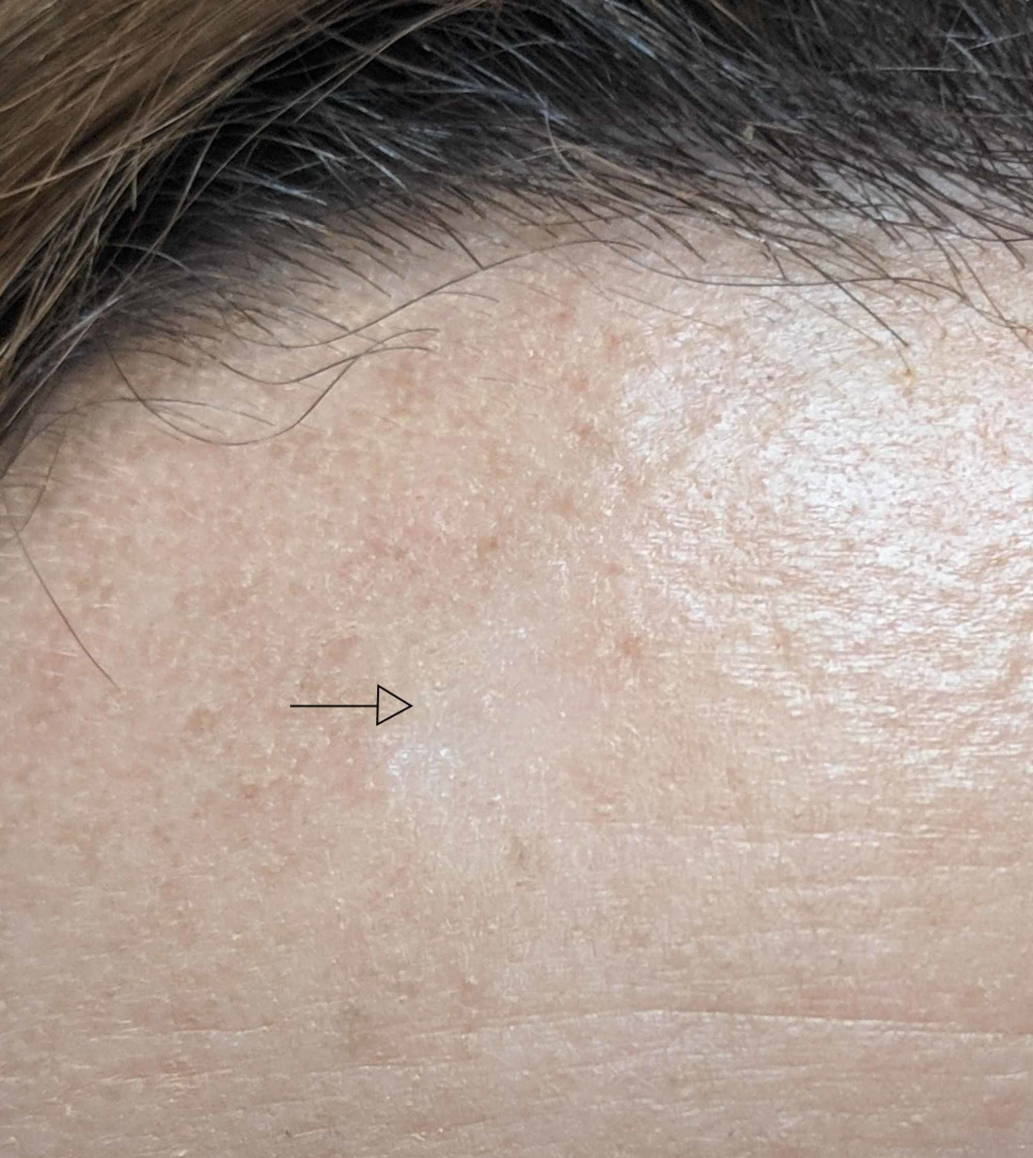
Right Superior Forehead Lesion A scaly, indurated plaque with faint peripheral erythema (arrow).

**Figure 2:**
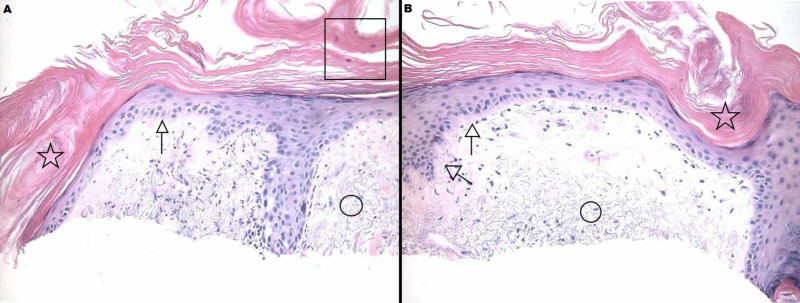
Right Superior Forehead Histology The shave biopsy shown in panels A and B demonstrates a thinned epidermis with thickening of the basement membrane zone (arrows), keratinous follicular plugging (stars), and ortho- and para-hyperkeratosis (square) of the epidermis. There is abundant dermal mucin throughout (circles).

A 35-year-old woman with past medical history significant for only chronic tobacco use presented for evaluation of an irritating lesion on her right forehead. Exam revealed an erythematous, scaly, indurated plaque on the right superior forehead (Figure 1). A biopsy was performed, and histology revealed ortho- and para-hyperkeratosis, an atrophic epidermis with sparse superficial inflammation, follicular plugging, thickening of the basement membrane zone, and abundant dermal mucin deposition (Figure 2). Complete blood count (CBC) and comprehensive metabolic panel were unremarkable, and an autoimmune panel was equivocal, revealing positive anti-double stranded DNA (dsDNA) and anti-Ro antibodies. Antinuclear antibody (ANA), anti-topoisomerase I (Scl70), anti-Smith (Sm), anti-ribonucleoprotein (RNP), and anti-La were negative. Intralesional steroids produced significant improvement of induration at one-month follow-up.

**Figure 3:**
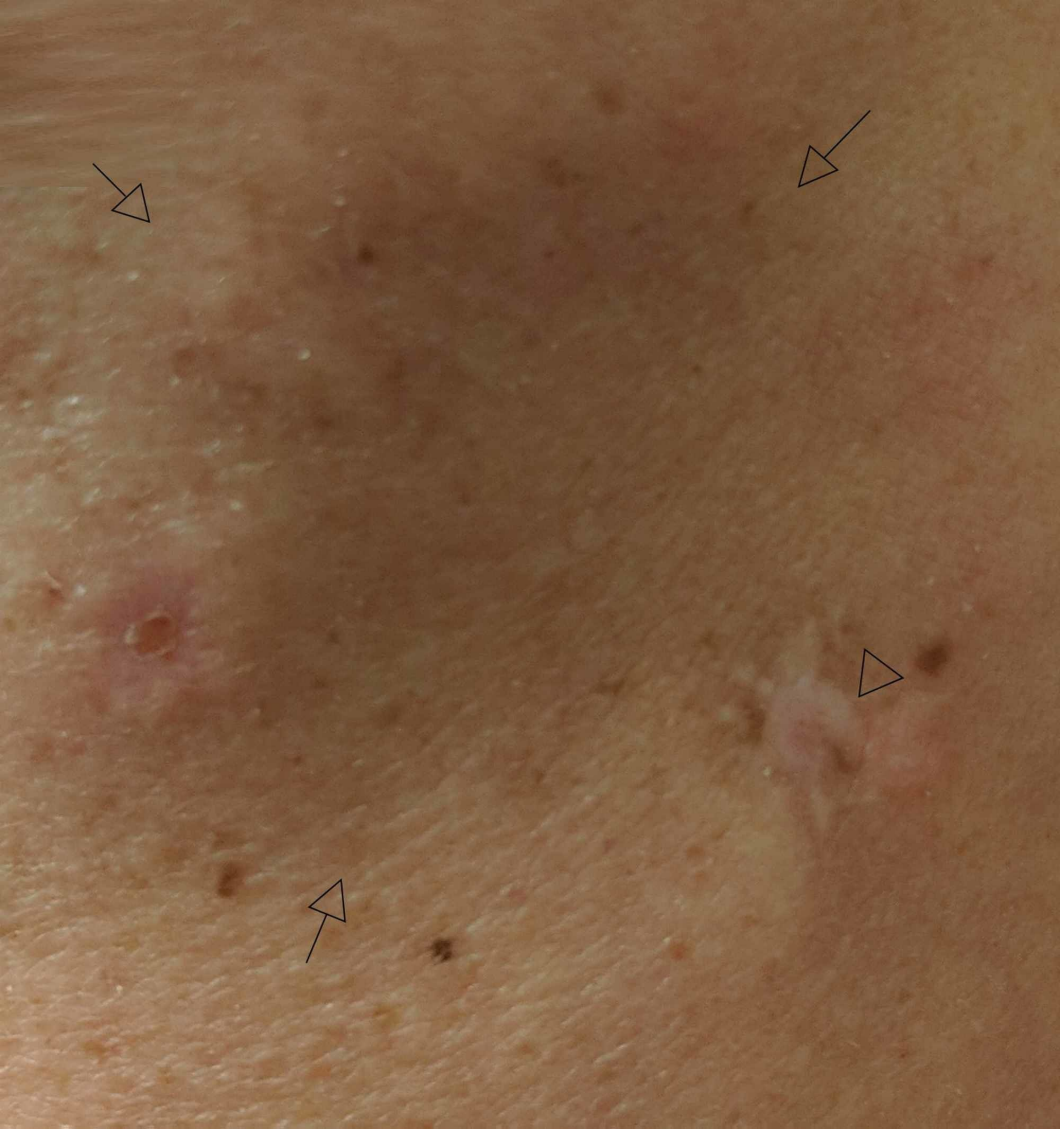
Left Posterior Arm Lesion Skin-colored, morpheaform plaque (arrows) with peripheral dyschromia (arrowhead).

**Figure 4:**
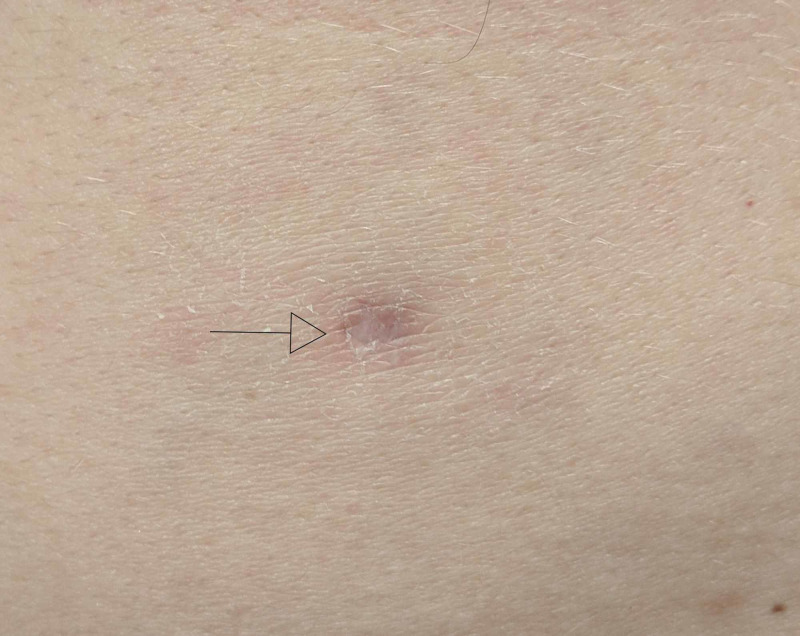
Left Flank Lesion An atrophic plaque with central hypopigmentation and moderate peripheral erythema (arrow).

Five months later, a new morpheaform plaque with a rim of dyschromia presented on the left posterior arm; the area was notably atrophic and significantly tender to palpation (Figure 3). An atrophic plaque with central hypopigmentation had also developed on the left flank, which was diagnosed clinically as DLE (Figure 4).

**Figure 5:**
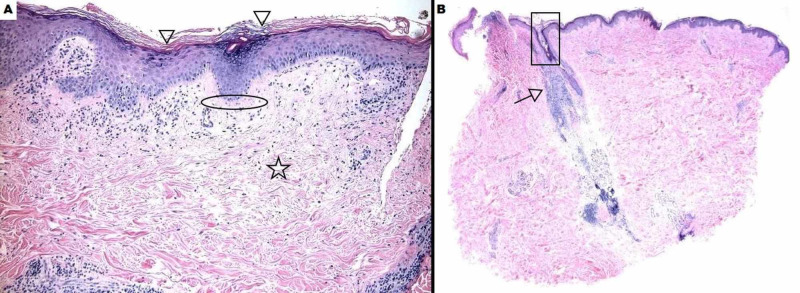
Left Posterior Arm Histology Displaying Discoid Lupus This punch biopsy section shows ortho-hyperkeratosis (arrow heads) with thickening of the basement membrane (oval) and dermal mucin (A). There is also follicular plugging (rectangle) and perifollicular inflammation (arrow) extending into the deep tissue (B).

**Figure 6:**
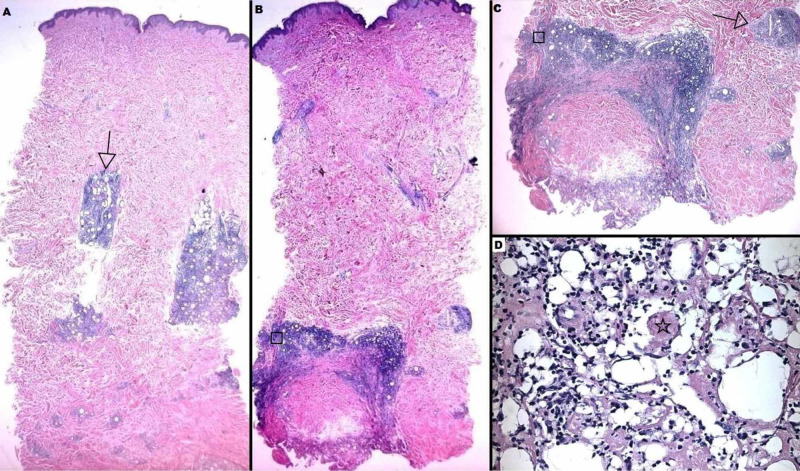
Left Posterior Arm Histology Displaying Lupus Panniculitis This section of the punch biopsy demonstrates relative sparing of the superficial aspect of the sample, with nodular lymphoid aggregates (arrows) in the deep dermis (A); focal lymphoplasmacytic inflammation (squares) surrounds the adipose tissue (B,C), with typical lymphocytes and areas of sclerosis (star) interspersed with degenerated adipocytes (D).

A telescoping punch biopsy was performed on the arm, which showed ortho-hyperkeratosis, thickening of the basement membrane zone, a superficial and deep lymphocytic infiltrate along the dermoepidermal interface and follicular structures, and pooling of dermal mucin (Figure 5); there were nodular lymphoid aggregates in the deep dermis and panniculus, with an intense lymphoplasmacytic infiltrate and abundant sclerosis surrounding degenerated adipocytes (Figure 6). These findings are consistent with DLE overlying LP. With our patient's progression to LP, we planned to initiate systemic hydroxychloroquine. 

The patient was strongly encouraged to refrain from tobacco use and was referred to ophthalmology for retinal evaluation and clearance to begin hydroxychloroquine therapy. The patient was also evaluated by rheumatology, who agreed with the decision to begin hydroxychloroquine and recommended biannual rheumatologic screenings to monitor for systemic disease involvement. Upon completing these consultations and seven months after initial presentation, the patient reported joint pains, recurrent oral ulcerations, and photosensitivity. With repeat ANA testing negative, the patient did not meet inclusion criteria for the European League Against Rheumatism (EULAR)/American College of Rheumatology (ACR) for SLE and thus could not be considered for diagnosis. However, the consulted rheumatologist considered our patient’s symptoms analogous to SLE, and continues to monitor her as such. She was started on hydroxychloroquine 200 mg twice daily. At one-year follow-up, the patient’s disease remained stable, with notable overlying atrophy of the LP affected sites but no inhibition of mobility. Joint pain and oral ulcerations had improved, and photosensitivity benefited from use of routine sunscreen. Overall, the patient was content with her status. 

## Discussion

DLE comprises an estimated 80% of all lupus-specific skin findings, while LP contributes just 1%-3% [3,4]. Both forms display a female predilection of roughly 3:1, with onset in the third to fifth decades, again aligning with our patient's demographics [5]. The diagnosis of DLE or LP carries a 5%-20% lifetime risk of developing SLE [6]. While LP most frequently occurs independently, it is seen with overlying discoid lesions in 10%-30% of cases and manifests alongside SLE 1%-3% of the time; approximately 10% of patients with DLE go on to develop LP, but it is unclear what factors contribute to this evolution [7-9].

A variety of environmental and genetic influences contribute to the complex interplay suspected to incite chronic cutaneous lupus. Ultraviolet radiation, medications, smoking, and viral elements are speculated to induce keratinocyte apoptosis and stimulate plasmacytoid dendritic cells, which amplify interferon signatures and excite the inflammatory cascade in genetically susceptible individuals [4,5,10]. An erroneous, lymphocyte-mediated immune response preferentially targets the basal epidermal layer, likely due to homing signals produced by specific cell ligands seen on keratinocytes and adnexa [11]. Several proteins, including intracellular adhesion molecule (ICAM), lymphocyte function-associated antigen 1 (LFA-1), chemokine ligand 17 (CCL17 or TARC), interleukin-18 (IL-18), and tumor necrosis factor (TNF), have been implicated [3,4]. Low complement 4 (C4) levels or preceding focal trauma have been correlated with the development of LP, specifically [12]. 

DLE most commonly presents on the face, scalp, and ears. Disseminated disease, with lesions above and below the neck, occurs in 20% of patients [1-3]. SLE will develop in 5% of patients with localized DLE and 20% in those with disseminated disease [2,3]. Mucosal and sun-protected sites may be affected, and are particularly difficult to diagnose because of the relatively unexpected presentation in these regions [8,9,13]. In our case, the patient's generalized DLE preceded the development of LP, likely correlating to a similar risk of more severe forms of CCLE, such as LP or recalcitrant DLE, being more common in disseminated disease.

Early DLE presents as an erythematous patch that often evolves into an atrophic, dull-white, dyspigmented plaque with hyperpigmented borders. Central scaling is characteristic, with follicular-based keratotic spikes that can be visualized if a plaque is pulled back, termed the carpet tack or cat’s tongue sign [3,13]. Scarring and follicular plugging leads to adnexal destruction, and resolved lesions heal with atrophy, telangiectasias, and pigmentary alteration, findings that are highlighted in our case figures [4,11].

LP favors densely lipomatous regions, such as the face and scalp, proximal extremities, buttocks, and trunk [2,5]. This chronic, relapsing condition often begins as deep, tender nodules in predilect areas. Eventual destruction of underlying adipose creates depressed plaques [3,14]. The term lupus profundus is reserved by some authors for instances of DLE occurring atop LP [5,8]. As adipose tissue destruction progresses, severe disfigurement and overlying ulceration become concerning risks [1,4]. Our patient complained of an exquisitely tender nodule prior to the atrophic change seen upon presentation; later, adipose damage lead to tethering of the skin to underlying structural components.

The clinical connection between LP and DLE has not been fully elucidated, and it remains unclear why up to 30% of patients with LP develop overlying DLE, while only 10% of patients with DLE develop underlying LP [7-9]. Protective or aggravating factors have not been firmly identified and, while it is generally accepted that LP requires more aggressive treatment than DLE, there are no recommendations for how to potentially interrupt progression of the spectrum of CCLE in those susceptible. We suspect that our patient's smoking history may have been a catalyzing factor, since tobacco use has been strongly linked to, typically recalcitrant, cutaneous lupus [4,5,10].

As in all cutaneous lupus, histologic assessment remains the gold standard for securing diagnoses of DLE and LP, with the inclusion of immunofluorescence in equivocal cases [1]. The model features seen in DLE include hyperkeratosis, keratotic follicular plugging, epidermal atrophy, and vacuolar interface changes leading to basement membrane thickening [1,4]. A dense lymphocytic inflammation of the adnexa and vasculature, dermal mucin deposition, and the presence of necrotic basement membrane cells termed civatte bodies are frequently identified [4,11].

Key findings seen in LP are a lobular lymphoplasmacytic panniculitis with hyaline fat necrosis, nodular lymphocytic aggregates, and mucin deposition [3,5,14]. Calcifications and thickened hyalinized vessels are common and prominent in subcutaneous regions [12]. The lymphocytic infiltrate can cause vasculitis and fibrinoid thrombosis, which may translate clinically into ulceration [1,4].

Direct immunofluorescence (DIF) aids in diagnosis in all cases of cutaneous lupus, as the presence of a continuous, granular band of immunoglobulins (Ig) G/A/M, and complement 3 (C3) along the dermoepidermal junction is a highly characteristic finding; however, IgM and C3 often predominate the deposit [4,8]. DIF studies of lesional skin are reportedly positive in 92% of ACLE, 60% of SCLE, and 80%-90% of CCLE [4].

Once the diagnosis of cutaneous lupus is confirmed, serologic testing is recommended to determine the presence and extent of systemic disease. Antinuclear antibodies are found with varying degree in cutaneous lupus, with positivity rates ranging from 95% in ACLE to 20%-66% in CCLE [4,10]. While specific autoantibodies rarely aid in differentiating cutaneous lupus subtypes, anti-Ro and anti-La antibodies are an exception, occurring almost exclusively in patients with SCLE [1]. Host antibodies against dsDNA and Smith are highly characteristic of SLE, occurring in 70% and 25% of cases, respectively; these findings are uncommon in primarily cutaneous disease but may relate to systemic, and particularly renal, disease [1,10]. Cutaneous lupus has a low incidence of positive ANA, dsDNA, Sm, or Ro/La antibodies, though the presence of anti-single-stranded DNA (ssDNA) in patients with DLE may confer a higher risk of developing SLE [4]. Due to the protean nature of SLE, practitioners may additionally screen suspected systemic lupus patients with a battery of serologic studies to rule out other connective tissue diseases.

With respect to SLE, it is important to note that the recently updated 2019 EULAR/ACR classification criteria improved upon the sensitivities and specificities of the previous 1997 ACR and 2012 Systemic Lupus International Collaborating Clinics (SLICC) [15]. One of the major changes within these recommendations uses ANA positivity as a requisite entry criterion to be considered for diagnosis, a position that is unsupported by the literature [16]. Aringer et al. identify the existence of ANA-negative patients and impart particular importance on investigating this population to produce an alternative criterion for this subset [15]. Our patient meets the 1997 ACR and 2012 SLICC criteria, but not the 2019 EULAR/ACR criteria, highlighting this limitation. This could impede her access to treatment modalities in the future, and underscores an important consideration when evaluating the proper utilization of classification criteria when diagnosing patients.

Sunscreen and smoking cessation are fundamental elements of managing all forms of CCLE, as both UV light and tobacco use have been identified as disease instigators [1,10]. Topical corticosteroids and calcineurin inhibitors also serve as first-line therapies for DLE, and demonstrate particular efficacy in acute, swollen, and non-hyperkeratotic lesions [2,8,17]. In unresponsive disease, intralesional corticosteroids followed by systemic antimalarials and retinoids are often prescribed [3,7]. The mainstays of treatment for LP are systemic antimalarials with oral corticosteroids often co-administered during induction and to combat acute flares [2,5,14]. In either case, hydroxychloroquine is widely preferred due to its accessibility and excellent safety profile, frequently dosed at 200-400 mg in divided daily doses, with a maximum recommended daily dosing of 6.5 mg/kg/day [7,17]. Our patient responded well to 200 mg of the drug twice daily, and, while her disease did not resolve, it remained stable.

Before initiating hydroxychloroquine (or chloroquine), it is important to have a baseline ophthalmologic evaluation to evaluate retinal health since these drugs may cause dose and duration-dependent retinopathy; the American Academy of Ophthalmology recommends subsequent evaluation after five years of therapy, and annually thereafter [18]. Maximal efficacy of antimalarials is typically seen within four to six weeks, which requires ample patient education and supports the inclusion of oral corticosteroids into the induction sequence. A second antimalarial, namely quinacrine, thalidomide or cytotoxic agents such as methotrexate, cyclosporine, or cyclophosphamide may be considered in resistant disease [6,10,17]. It is critical that the provider monitors the patient for any unique adverse effects related to any infrequently used therapy.

The courses of DLE and LP are unpredictable, and most patients will experience cyclic exacerbation and remission [11]. Early identification and treatment are essential in limiting the local destruction, scarring, and dyspigmentation that develop with unbridled disease. Routine follow-up of any cutaneous lupus variant is recommended to ensure proper treatment is utilized and that evolution of disease is rapidly recognized and arrested.

## Conclusions

It is important to recognize the wide spectrum of CCLE. There exists sparse literature regarding the progression of DLE to LP, either in the context of evolving individual lesions or the development of more serious forms of CCLE in the same patient. While various studies have reported the concurrent rates of these dermatologic findings, there have been no specific factors identified that can be used to predict or inhibit the development of LP. Despite the fact that DLE and LP are often recalcitrant conditions, the disease processes and presentations are quite different and, thus, there remains tremendous utility in discerning between the two. Early detection of LP affords proper advancement to systemic steroid and antimalarial therapies, with the hope of limiting or even avoiding the disfiguring subcutaneous atrophy that often results from active disease. These concerns demand that the clinician monitors cutaneous lupus patients closely for evidence of new or progressive symptoms.

This case represents the development of DLE with subsequent progression to LP and, likely, systemic lupus. This evolutionary process is underrecognized and unexplained by the current literature. Due to the chronicity of cutaneous lupus, it is necessary that clinicians recognize the presentation and risk factors for this entity and, specifically, identify signs that correlate with evolution of DLE to LP. This will allow proper treatment modalities to be enacted, limiting the ill effects of disease.
